# Grad-CAM-Based Explainable Artificial Intelligence Related to Medical Text Processing

**DOI:** 10.3390/bioengineering10091070

**Published:** 2023-09-10

**Authors:** Hongjian Zhang, Katsuhiko Ogasawara

**Affiliations:** Graduate School of Health Science, Hokkaido University, N12-W5, Kitaku, Sapporo 060-0812, Japan

**Keywords:** explainable artificial intelligence (XAI), text processing, Grad-CAM, ResNet

## Abstract

The opacity of deep learning makes its application challenging in the medical field. Therefore, there is a need to enable explainable artificial intelligence (XAI) in the medical field to ensure that models and their results can be explained in a manner that humans can understand. This study uses a high-accuracy computer vision algorithm model to transfer learning to medical text tasks and uses the explanatory visualization method known as gradient-weighted class activation mapping (Grad-CAM) to generate heat maps to ensure that the basis for decision-making can be provided intuitively or via the model. The system comprises four modules: pre-processing, word embedding, classifier, and visualization. We used Word2Vec and BERT to compare word embeddings and use ResNet and 1Dimension convolutional neural networks (CNN) to compare classifiers. Finally, the Bi-LSTM was used to perform text classification for direct comparison. With 25 epochs, the model that used pre-trained ResNet on the formalized text presented the best performance (recall of 90.9%, precision of 91.1%, and an F1 score of 90.2% weighted). This study uses ResNet to process medical texts through Grad-CAM-based explainable artificial intelligence and obtains a high-accuracy classification effect; at the same time, through Grad-CAM visualization, it intuitively shows the words to which the model pays attention when making predictions.

## 1. Introduction

In recent years, artificial intelligence (AI) technology has made significant progress and become more complex and sophisticated. With the rapid increase in the data volume and improvements in computer performance, the development of AI has ushered in huge improvements. AI has achieved practical applications in several fields, such as speech recognition, automatic driving, and recommendation systems [1,2,3,4]. In the medical field, AI can improve people’s health by improving doctors’ diagnostic efficiency, intensive preventive treatment, and personalized recommendations for electronic medical records. Although many useful technologies have been applied in the medical field, the opacity of machine-learning and deep learning algorithms in AI poses ethical and practical challenges for their application. Without an understanding of AI diagnoses, it is impossible to determine whether patient diagnostic differences reflect important diagnostic differences owing to biases or diagnostic errors. AI’s key goal in medicine is to provide personalized advice for personalized medicine, healthcare, and drug selection. However, the current state of the application of AI in medicine has been described using the phrase “promising but lacks evidence and proof.” In real clinical settings, AI has been tested for its ability to detect diseases, such as clavicle fractures, diabetic retinopathy, cancer metastasis, and breast cancer. However, many systems that have demonstrated comparable or better performance than experts in these experiments exhibit high false-positive rates in real-world clinical settings [5]. Simultaneously, there are issues regarding discrimination, security, privacy, and a lack of transparency, which are related to trust, fairness, messaging, enforceability, and causal inference [6].

Therefore, the concept of explainable artificial intelligence (XAI) was proposed. XAI emphasizes that AI should have explainability, meaning that the model and its results can be explained in a way that humans can understand. XAI allows the interpretation of the learned model and analyzes its logical flow by focusing on the reasons that the system has a given demerit [7]. Simultaneously, a growing number of experts at the intersection of AI and healthcare have concluded that the ability of AI models to provide explanations to humans is more important than their accuracy when it comes to practical applications in clinical settings. It is important to understand the way in which they operate before adopting and using medical AI applications. Therefore, medical XAI is necessary to ensure the acceptability of AI for blocking applications in medicine.

In XAI, gradient-weighted class activation mapping (Grad-CAM) is a visualization method. This method provides explainability by visualizing the area on which the model focuses when making predictions in the form of a heat map, intuitively showing the basis used by the model to make decisions. Compared to the CAM method, this method can be applied to various convolutional neural networks (CNN) without adjusting the network structure and has better versatility. This approach is now widely used in computer vision-related tasks to intuitively obtain model interpretability. Therefore, this study considers applying this concept to related word-processing tasks to ensure that text-processing tasks can also achieve interpretable visualization.

The impact of AI has been widely discussed [8,9,10]. AI can be used to develop complex algorithms to “read” features of a large amount of healthcare data and use the learned knowledge to help clinical practice. To improve accuracy based on feedback, AI can be equipped with learning and self-correction functions. By presenting the latest medical knowledge from journals, manuals, and professional procedures to provide recommendations for delivering effective patient care, AI devices [11] can support clinical decision-making. In addition, in human clinical practice, AI systems may help to reduce inevitable medical and treatment errors (i.e., they are more objective and repeatable) [8,9,10,11,12,13,14,15]. In addition, to facilitate real-time inferences of health risk warnings and health outcome estimates, AI systems can process valuable knowledge gathered from a large population of patients [16].

With the recent re-entry of AI into science and public awareness, there have been new breakthroughs in AI in the field of healthcare, and the clinical environment is being filled with new AI technologies at an extremely fast rate. Nevertheless, healthcare is one of the most exciting applications of AI. Since the 20th century, researchers have proposed and established multiple systems to support clinical decisions [17,18]. Since the 1970s, the rule-based approach has achieved many successes and is now seen as a theory that explains electrocardiograms [19], identifies diseases [20], chooses the best therapy [21], provides scientific logical explanations [22], and assists doctors in formulating diagnostic hypotheses and challenging patient cases [23]. However, rule-based systems are expensive and unstable because they need to clearly express decision-making rules and, like textbooks, require manual modifications. In addition, high-level interactions among various pieces of information compiled by different experts are difficult to code, and the efficiency of the structure is limited by the comprehensiveness of prior medical knowledge [24]. It is difficult to combine deterministic and probabilistic reasoning procedures to narrow down the appropriate psychological background, prioritize medical theories, and prescribe treatment plans; it is also difficult to create a method that combines deterministic and probabilistic reasoning procedures [25,26].

Compared to general text, medical text has features such as mass terminology and contains structured text data and free-text data. In the medical textual processing field, AI is mainly used to deal with the tasks of text classification, namely entity recognition and relationship extraction [27]. During text classification, because of the information contained in text data, text classifiers based on deep neural networks have fewer layers and their performance is slightly inferior than that of the mature and high-accuracy methods of image classifiers.

This study uses a high-accuracy computer vision algorithm model to transfer learning to medical text tasks while simultaneously using the interpretive visualization method Grad-CAM to generate heat maps to ensure that the basis for decision-making can be provided intuitively or via the model. This study makes the black-box neural network model interpretable by attaching the Grad-CAM module to the model and can be visualized through the heatmap. In order to generate the heat map, a CNN method is required, meaning that this study extracts features from the text by performing word embedding and presents the text as an image (the dimensions of the word vectors replace the channels of the RGB image). And, unlike previous works that treated text as a one-dimensional vector format, this study keeps the text in a two-dimensional structure similar to that of an image, maximizing the utilization of spatial information via the CNN method.

## 2. Materials and Methods

This study used models that are often used in computer vision area to transfer learning to text-processing tasks and used the Grad-CAM method to visualize the explainability of the model. The modules used in this study were pre-processing, word-embedding, classification, and visualization modules. Word2vec was used as the word-embedding tool, and ResNet was used as the main classifier. Clinical text data derived from an open dataset containing reports of five classes (digestive system, cardiovascular system, neoplasm, nervous system, and general pathology) were used.

Grad-CAM is an interpretable method derived from computer vision [28]. For CNNs, this method is a well-established ex-post hoc explainable technique. Furthermore, the Grad-CAM method passes an independent sanity check [29]. Although it is primarily related to the interpretation of deep-learning networks used for image data, it has also been adapted to other application domains. CNN architectures used in text classification are described in [30], and at least one implementation that extends this work exists to support Grad-CAM and interpretability [31]. Grad-CAM is used in the NLP domain to perform document retrieval [32].

### 2.1. System Architecture

**Figure 1:**
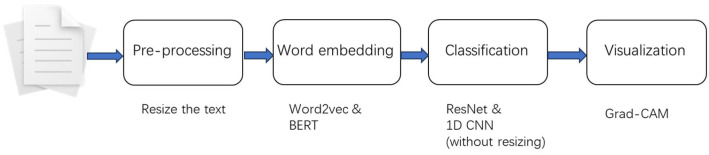
Structure of study.

The system structure is shown in Figure 1, including the following four modules: a pre-processing module, which aims to convert each text record into a specific format; a word-embedding module, which aims to convert each word in the text into a word vector; a classifier module, which classifies text through a transfer learning image-processing method; and a visualization module, which aims to mark and display the areas to which the model pays attention when making predictions.

The output of the pre-processing module was fed into the word-embedding module. The word embeddings used were variants of Word2Vec and BERT. These two word-embedding modes were selected because they represent order-independent and order-dependent word-embedding modes, respectively. Word2vec takes the form of a bag of words, and it is insensitive to the order of the words entered. BERT is an encoder that uses a transformer and assumes that the word order of an input sentence has a meaning.

In the classification module, the output of the word-embedding module was fed into a tuned ResNet network. Although ResNet as a CNN architecture has its origins in computer vision, where images form the input to the network, it is reasonable to use a sequence of word vectors as the input. In a sentence, the relative positions of words convey their meanings. This approach is similar to the role played by the pixels in conveying information in an image. Therefore, in the pre-processing module used in this study, the text was adjusted to a two-dimensional format to make it similar to an image. In addition, the image content is usually 3-channel (RGB) or single-channel (binary or grayscale) in nature. This study uses word vectors as the input, and each element of the word vector is used as the value of a channel, meaning that it will be used as the classifier. ResNet is adjusted to ensure that it can accept the channel of the number of word–vector dimensions (100 and 256 in this study). And this study also used the AlexNet and VGG11 as classifiers to perform comparisons. Simultaneously, a one-dimensional CNN was prepared to classify sentences without text pre-processing to compare the effects of the pre-processing module.

The visualization module uses the Grad-CAM technology to generate class activation maps (thermal phase maps) for a given text and predicted class. Each element of the class activation map corresponds to a token and indicates its importance based on the score of a particular class. Class activation maps provide information regarding the extent to which a particular token present in an input sentence affects ANN predictions.

The software stack used to carry out development was Python version 3.8.3. PyTorch was used for ResNet and Grad-CAM. The word2vec model was derived from Gensim.

### 2.2. Experiment

#### 2.2.1. Dataset

We used the open medical text dataset in Kaggle, which contains 14,438 clinical texts divided into five categories. This dataset consists of medical abstracts that describe the current condition of a patient. And this dataset was described as a situation in which doctors routinely scan dozens or hundreds of abstracts each day as they complete their duties in a hospital, meaning that they must quickly pick up on the salient information pointing to the patient’s malady. This study used this dataset to classify the class of problems described in these abstracts. All of the texts have been tagged. One-fifth of the training set was used as the verification set; its categories and quantities are listed Table 1.

#### 2.2.2. Pre-Processing

To better apply the image-processing algorithm, each record in the dataset was converted into a 25 × 25 (number of words) format. This size format was used because the maximum text length in the dataset was 600; therefore, a size of 25 × 25 = 625 was chosen to accommodate all of the text. If the number of words in the text was insufficient, the # symbol was used.

#### 2.2.3. Word Embedding

After using Word2Vec to generate word vectors on the training set without pre-processing, the window size was 100, and the generated word-vector dimension was 100. The word vectors of the filled symbols and unrecognized words are set as zero vectors. The BERT model uses BERT-mini, with parameters L = 4 and H = 256 denoted as the word-embedding method.

#### 2.2.4. Classification and Visualization

**Figure 2:**
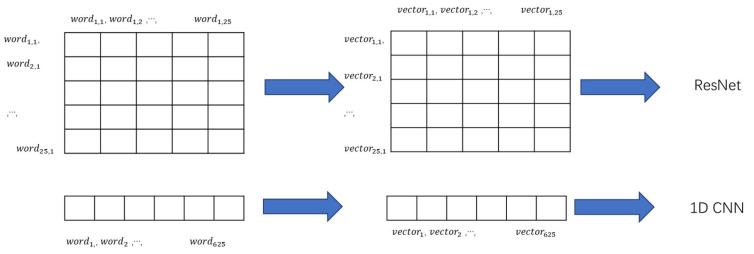
Process of text classification.

ResNet and 1D CNN were used as classifiers to perform text classification. For ResNet, we used ResNet18 with the pre-trained parameters. In addition, each text in the 25 × 25 format was treated as a picture with several channels in the word–vector dimension, that is, the m elements of the word in the jth row and the kth column in text i corresponded to the point p of picture i on channel m (j,k). Simultaneously, the model was fine-tuned such that the input channel was the word–vector dimension and the output size was 5. For the 1D CNN, LeNet was modified into a one-dimensional structure. The input text was not formatted as 25 × 25, instead using a one-dimensional text vector column, and was filled up to a length of 625. The process used is shown in Figure 2. In addition, the Naïve Bayes method of the traditional text classifier was used to perform comparison, using tf-idf as the feature extraction, text without word embedding and length padding as the input. Finally, a Grad-CAM module was added to the classifier using the CNN method to visualize the attention points of the model during the classification process and display them in the form of a heat map. The parameters and settings for each model are listed in Table 2.

## 3. Results

Table 3 presents the performance of each model using recall, precision, F1, and accuracy as indicators. As the data used in this study were biased in terms of the amount of data in each category, weighted recall, precision, and F1 values were used to perform the evaluation. The 2D form of ResNet18 using pre-trained parameters and re-training on the Kaggle medical text dataset exhibited the best performance, having a weighted F1 value of 90.2% and an accuracy rate of 90.9%. Simultaneously, the weighted F1 values of the traditional text classifier Naïve Bayes model used to perform comparison were 42.2% and 47.8%, respectively. And the models of AlexNet and VGG11 did not have a good performance, as the accuracy rates were lower than that of Naïve Bayes.

Table 4 lists the differences in the effects of ResNet18 (word embedding through word2vec) in different situations. The compared models were as follows: (1) fine-tuning the input and output layers of ResNet, using the parameters pre-trained on ImageNet and re-training the model on the medical text dataset, that is, the model shown in Table 2 in this study; (2) only the input and output layers of ResNet were fine-tuned, and the model was trained using the medical text dataset; (3) the input and output layers of ResNet were fine-tuned, and the parameters were pre-trained in ImageNet. The above three models selected the model with the best performance in the case of Epoch 25. In the case of direct training without using pre-training parameters, the F1 value was only 40.2%, and the correct rate was only 46%, mainly because it had not yet converged at Epoch 25 and the training of the model was insufficient. In the case of only using the pre-training parameters of ImageNet, the performance was extremely poor (F1 7.7%, accuracy 12.5%) because the parameters were trained using ImageNet images, and the input was three channels; this study used data in a 100-channel format as the input, which did not match its parameters.

**Figure 3:**
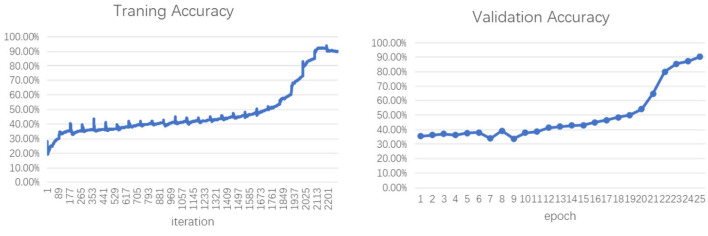
Accuracy of ResNet18 in training and validation.

Figure 3 shows the training and verification accuracies of the ResNet18 model over Epoch 25. The left and right figures show the training and verification processes, respectively. The horizontal axis of the training process represents the number of iterations, and one epoch of the training process contained 91 iterations. The ResNet18 model converged and, finally, found an accuracy of over 90% in training and validation.

**Figure 4:**
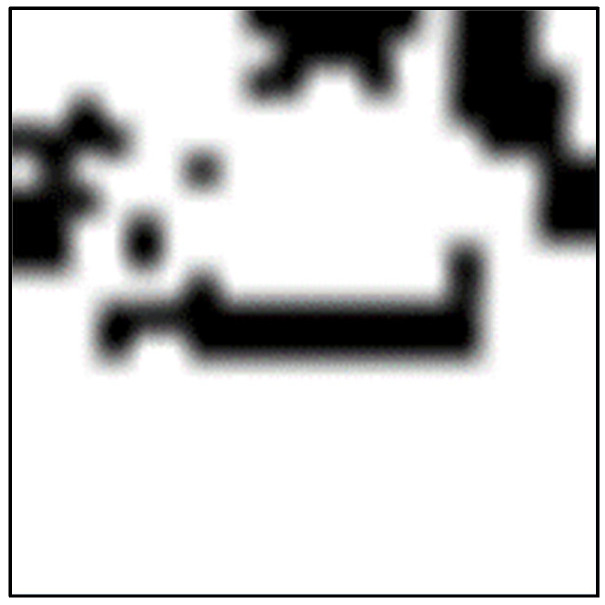
Example of a heatmap used to predict text of the digestive system.

**Figure 5:**
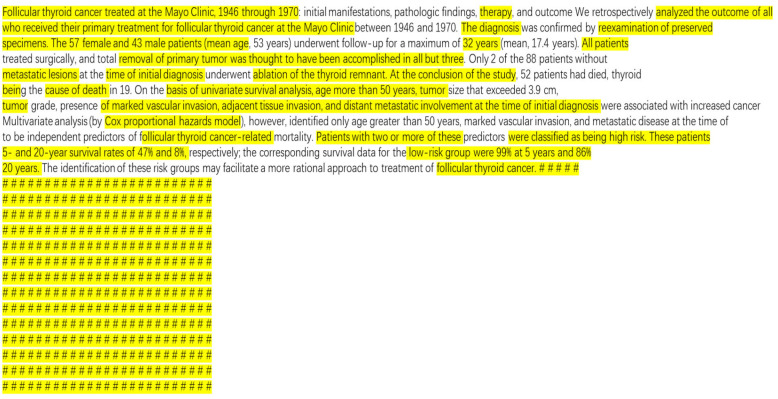
Highlighting the position of the text combined with the heatmap.

Figure 4 shows an example of a heatmap generated for use in Grad-CAM, indicating the regions on which the model focuses when making predictions. The white area indicates that the attention of the model is high. Figure 5 shows the text corresponding to the heat map shown in Figure 3. The example shows the prediction of the text of the digestive system and highlights the position on which the model focused during the prediction. As shown in the figure, the model focuses on “follicular thyroid cancer treated at the Mayo Clinic, 1946 through 1970.”

## 4. Discussion

This study applied ResNet to medical text-processing tasks and demonstrated the interpretability of the model using Grad-CAM. In the field of medical texts, Grad-CAM has not been used to generate heat maps to achieve interpretable AI. This study used Grad-CAM to generate heat maps, which visually present the words to which the model pays attention when making predictions and realizes the interpretability of the model. Compared to the research on the use of Grad-CAM in the legal field [33], this study formatted the text into a two-dimensional form and used the more mature CNN technology instead of directly using 1D CNN to process the text; making greater use of the advantages of CNN, it has better results.

Compared to other XAI methods, Grad-CAM can visualize the explainability of a model and be easily added to various ANN methods to visualize the model interpretation. Other interpretable methods for text, such as knowledge graphs, ontology databases, and other interpretable methods used to construct rules, incur considerable labor costs in construction and updating. As a post hoc XAI method, Grad-CAM has a higher degree of freedom, and its structure can be adapted to various ANN methods. Even if the model was modified, it could be processed using the same procedure. In contrast, Grad-CAM can only provide explainability but not interpretability, that is, it can only determine the basis of the results given by the model, and it cannot know the reasoning process influencing the results given by the model. Thus, this study depends on the work of clinical doctors to determine whether the explainablity given by the model is suitable or not.

Kilimci et al. confirmed that deep-learning methods perform better than traditional methods in text classification [34]. However, these studies directly applied deep neural networks to the text and trained the features obtained via extracting the tf-idf or word vector of the text as a feature. This approach means that these black box models have low interpretability regarding the conclusions drawn. Some studies directly apply deep learning CNN to text classification and achieve a certain degree of interpretability by mixing the word’s word vector with the clinical identifier CUI corresponding to the word [35]. This study shows that models using deep learning have comparable or even better performances than traditional methods. However, the deep learning model implemented by mixing word vectors was slightly less intuitive for interpreting the results. This study considered converting text information into an image format, which was convenient for attaching additional explanation modules, and the interpretation of the results was more intuitive [36].

In addition, this study explored the transfer learning of ResNet, which is commonly used in image processing and text processing, and compared it to traditional text-processing methods, such as Naïve Bayes and Bi-LSTM. Compared to other deep learning networks, ResNet presented a superior performance, as its structure can solve the problem of gradient explosion. At the same time, it is compared to the results of directly using the 1D CNN in the classifier module to verify the advantages and disadvantages of converting the text format into a two-dimensional form. Previous studies [33] tended to use a 1D CNN to directly process text sequences. And as a limitation, this study did not compare the performance to those of some state-of-the-art models, but only showed the performance of the proposed model and compared it to some classical models. The comparison with state-of-the-art models will be demonstrated in a future work.

Text was used in the image format to transfer learning using the image-processing model, and Grad-CAM was used to realize and visualize the interpretation of the model. The limitation is that the interpretability displayed via Grad-CAM is qualitative, rather than quantitative, in nature. Although this method shows the basis of the model, it cannot determine whether the basis is good or bad. This issue can only be judged based on a doctor’s observations of the model. A subjective evaluation of the position was used to evaluate the reliability of the model when making judgments. Future research should consider criteria used to measure the quality of model interpretability. The criteria SHapley additive explanation (SHAP) and locally interpretable model agnostic explanations (LIME) were used in the research of Naresh Kumar et.al [37]. SHAP can be used to describe whether an input feature is present in the model or not. And LIME can be used by the classifier to identify explicit individual prediction scores. These metrics might be used in future works to measure the explainability. And as mentioned in the research of Yuchen Jiang et.al [38], XAI needs to outline which factors contribute most to the results and the best way to interpret results using linguistic expressions. For the “black-box” models such as ANNs, it is difficult to interpret the process of the results identified via linguistic expression. Thus, XAI is bound to focus on indicating the sub-blocks of the deep structures though ablation experiments. In this way, the factors contribute to the results, as can be explicated. This study depends on the work of clinical doctors to estimate whether the factors contributing to the result are suitable or not. And these objectives will be the direction of future work.

## 5. Conclusions

ResNet was used to perform medical text processing using interpretable AI based on Grad-CAM. The results were as follows:

1. We used ResNet to perform text processing and obtain a higher accuracy classification effect;
2. The Grad-CAM visualization intuitively showed the words to which the model paid attention when making predictions and realized the interpretability of the model.

The problem that needs to be solved in the future is that this study used a qualitative method. The method used Grad-CAM to generate heatmaps, and doctors judged whether the result was right or not depending on the heatmap. In the future, we will seek a criteria to quantitatively measure the explainability found via Grad-CAM, and we will compare the performance to those of some state-of-the-art models.

## Figures and Tables

**Table 1:** Number of reports in each category.

	Training	Validation
Digestive system	2530	632
Cardiovascular	1194	299
Neoplasms	1539	385
Nervous system	2440	610
General pathological	3843	961

**Table 2:** Settings of models.

Models	Parameters	Value
Word2vec	Vector size	100
	Model	Skip-gram
	Optimizer	Hierarchical softmax
	Window size	100
	Min_count	1
ResNet18	Learn rate	0.1
	Loss	CrossEntropy
	Optimizer	SGD
		Weight decay 5e-4
	Batch size	128
1 D CNN	Learn rate	0.1
	Loss	CrossEntropy
	Optimizer	SGD
		Weight decay 5e-4
	Batch size	128
Naïve Bayes	Feature extraction	Tf-idf

**Table 3:** Performance of each model.

	Recall (Weighted)	Precision (Weighted)	F1 Score (Weighted)
Naïve Bayes	47.8	62.8	42.2
Bi-LSTM	74.4	77.9	76.2
ResNet (Word2Vec)	90.9	91.1	90.2
1D CNN (Word2Vec)	10.0	1.0	8
AlexNet (Word2Vec)t	32.6	10.6	16.0
VGG11 (Word2Vec)	32.6	10.6	16.0
ResNet (BERT)	85.7	87.9	86.8
1D CNN (BERT)	12.0	2.3	8.7

**Table 4:** Performance of different models on ResNet(Word2Vec).

	Recall (Weighted)	Precision (Weighted)	F1 Score (Weighted)
With fine-tuning and pre-trained parameters	90.9	91.1	90.2
With fine-tuning but no pre-trained parameters	46.0	42.4	40.2
Only with fine-tuning	12.5	10.8	7.7

## Data Availability

The datasets (https://www.kaggle.com/datasets/chaitanyakck/medical-text (accessed on 28 June 2023)) related to this study is publicly available.

## References

[1] Gupta A., Anpalagan A., Guan L., Khwaja A.S. (2021). Deep learning for object detection and scene perception in self-driving cars: Survey, challenges, and open issues. Array.

[2] Bharati S., Podder P., Mondal M.R.H. (2020). Artificial Neural Network Based Breast Cancer Screening: A Comprehensive Review. Int. J. Comput. Inf. Syst. Ind. Manag. Appl..

[3] Mondal M.R.H., Bharati S., Podder P. (2021). CO-IRv2: Optimized InceptionResNetV2 for COVID-19 detection from chest CT images. PLoS ONE.

[4] Bharati S., Podder P., Mondal M.R.H. (2020). Hybrid deep learning for detecting lung diseases from X-ray images. Inform. Med. Unlocked.

[5] Topol E.J. (2019). High-performance medicine: The convergence of human and artificial intelligence. Nat. Med..

[6] Arrieta A.B., Díaz-Rodríguez N., Del Ser J., Bennetot A., Tabik S., Barbado A., García S., Gil-López S., Molina D., Benjamins R. (2020). Explainable Artificial Intelligence (XAI): Concepts, taxonomies, opportunities and challenges toward responsible AI. Inf. Fusion.

[7] Vishwarupe V., Joshi P.M., Mathias N., Maheshwari S., Mhaisalkar S., Pawar V. (2022). Explainable AI and Interpretable Machine Learning: A Case Study in Perspective. Procedia Comput. Sci..

[8] Dilsizian S.E., Siegel E.L. (2014). Artificial intelligence in medicine and cardiac imaging: Harnessing big data and advanced computing to provide personalized medical diagnosis and treatment. Curr. Cardiol. Rep..

[9] Patel V.L., Shortliffe E.H., Stefanelli M., Szolovits P., Berthold M.R., Bellazzi R., Abu-Hanna A. (2009). The coming of age of artificial intelligence in medicine. Artif. Intell. Med..

[10] Jha S., Topol E.J. (2016). Adapting to artificial intelligence: Radiologists and pathologists as information specialists. JAMA.

[11] Strickland E. (2019). IBM Watson, heal thyself: How IBM overpromised and underdelivered on ai health care. IEEE Spectr..

[12] Weingart N.S., Wilson R.M., Gibberd R.W., Harrison B. (2000). Epidemiology of medical error. BMJ.

[13] Graber M.L., Franklin N., Gordon R. (2005). Diagnostic error in internal medicine. Arch. Intern. Med..

[14] Winters B., Custer J., Galvagno S.M., Colantuoni E., Kapoor S.G., Lee H., Goode V., Robinson K., Nakhasi A., Pronovost P. (2012). Diagnostic errors in the intensive care unit: A systematic review of autopsy studies. BMJ Qual. Saf..

[15] Lee C.S., Nagy P.G., Weaver S.J., Newman-Toker D.E. (2013). Cognitive and system factors contributing to diagnostic errors in radiology. Am. J. Roentgenol..

[16] Neill D.B. (2013). Using artificial intelligence to improve hospital inpatient care. IEEE Intell. Syst..

[17] Miller R.A. (1994). Medical diagnostic decision support systems—Past, present, and future: A threaded bibliography and brief commentary. J. Am. Med. Inform. Assoc..

[18] Musen M.A., Middleton B., Greenes R.A. (2014). Clinical decision-support systems. Biomedical Informatics.

[19] Kundu M., Nasipuri M., Basu D.K. (2000). Knowledge-based ECG interpretation: A critical review. Pattern Recognit..

[20] De Dombal F., Leaper D., Staniland J.R., McCann A., Horrocks J.C. (1972). Computer-aided diagnosis of acute abdominal pain. Br. Med. J..

[21] Shortliffe E.H., Davis R., Axline S.G., Buchanan B.G., Green C.C., Cohen S.N. (1975). Computer-based consultations in clinical therapeutics: Explanation and rule acquisition capabilities of the MYCIN system. Comput. Biomed. Res..

[22] Barnett G.O., Cimino J.J., Hupp J.A., Hoffer E.P. (1987). DXplain: An evolving diagnostic decision-support system. JAMA.

[23] Miller R.A., McNeil M.A., Challinor S.M., Masarie F.E., Myers J.D. (1986). The internist-1/quick medical reference project—Status report. West. J. Med..

[24] Berner E.S., Webster G.D., Shugerman A.A., Jackson J.R., Algina J., Baker A.L., Ball E.V., Cobbs C.G., Dennis V.W., Frenkel E.P. (1994). Performance of four computer-based diagnostic systems. N. Engl. J. Med..

[25] Szolovits P., Pauker S.G. (1978). Categorical and probabilistic reasoning in medical diagnosis. Artif. Intell..

[26] Szolovits P., Pauker S.G. (1994). Categorical and probabilistic reasoning in medicine revisited. Artificial Intelligence in Perspective.

[27] Wu S., Roberts K., Datta S., Du J., Ji Z., Si Y., Soni S., Wang Q., Wei Q., Xiang Y. (2020). Deep learning in clinical natural language processing: A methodical review. J. Am. Med. Inform. Assoc. JAMIA.

[28] Selvaraju R.R., Cogswell M., Das A., Vedantam R., Parikh D., Batra D. (2019). Grad-cam: Visual explanations from deep networks via gradient-based localization. Int. J. Comput. Vis..

[29] Adebayo J., Gilmer J., Muelly M., Goodfellow I., Hardt M., Kim B., Bengio S., Wallach H., Larochelle H., Grauman K., Cesa-Bianchi N., Garnett R. (2018). Sanity checks for saliency maps. Advances in Neural Information Processing Systems.

[30] Kim Y. Convolutional neural networks for sentence classification. Proceedings of the 2014 Conference on Empirical Methods in Natural Language Processing (EMNLP).

[31] Grad-Cam for Text. https://github.com/HaebinShin/grad-cam-text.

[32] Choi J., Choi J., Rhee W. (2020). Interpreting neural ranking models using grad-cam. arXiv.

[33] Gorski L., Ramakrishna S., Nowosielski J.M. (2020). Towards grad-cam based explainability in a legal text processing pipeline. arXiv.

[34] Kilimci Z.H., Akyokus S. (2018). Deep learning-and word embedding-based heterogeneous classifier ensembles for text classification. Complexity.

[35] Wang P., Kong X., Guo W., Zhang X. (2021). Exclusive Feature Constrained Class Activation Mapping for Better Visual Explanation. IEEE Access.

[36] Yao L., Mao C., Luo Y. (2019). Clinical text classification with rule-based features and knowledge-guided convolutional neural networks. BMC Med. Inform. Decis. Mak..

[37] Kumar N., Sharma M., Singh V.P., Madan C., Mehandia S. (2022). An empirical study of handcrafted and dense feature extraction techniques for lung and colon cancer classification from histopathological images. Biomed. Signal Process. Control.

[38] Jiang Y., Li X., Luo H., Yin S., Kaynak O. (2022). Quo Vadis Artificial Intelligence?. Discov. Artif. Intell..

